# A novel model for predicting prognosis and response to immunotherapy in nasopharyngeal carcinoma patients

**DOI:** 10.1007/s00262-023-03626-w

**Published:** 2024-01-18

**Authors:** Ya-Xian Wu, Bo-Yu Tian, Xin-Yuan Ou, Meng Wu, Qi Huang, Run-Kun Han, Xia He, Shu-Lin Chen

**Affiliations:** 1 Department of Clinical Laboratory, State Key Laboratory of Oncology in South China, Guangdong Key Laboratory of Nasopharyngeal Carcinoma Diagnosis and Therapy, Guangdong Provincial Clinical Research Center for Cancer, Sun Yat-Sen University Cancer Center, 651 Dongfeng Road East, Guangzhou, 510006 Guangdong People’s Republic of China; 2 Research Center for Translational Medicine, First Affiliated Hospital, Sun Yat-Sen University, Guangzhou, 510006 People’s Republic of China

**Keywords:** Immune checkpoint inhibitors, Nasopharyngeal carcinoma, Predictive biomarkers, Lasso Cox regression analysis

## Abstract

Blood-based biomarkers of immune checkpoint inhibitors (ICIs) response in patients with nasopharyngeal carcinoma (NPC) are lacking, so it is necessary to identify biomarkers to select NPC patients who will benefit most or least from ICIs. The absolute values of lymphocyte subpopulations, biochemical indexes, and blood routine tests were determined before ICIs-based treatments in the training cohort (*n* = 130). Then, the least absolute shrinkage and selection operator (Lasso) Cox regression analysis was developed to construct a prediction model. The performances of the prediction model were compared to TNM stage, treatment, and Epstein–Barr virus (EBV) DNA using the concordance index (C-index). Progression-free survival (PFS) was estimated by Kaplan–Meier (K–M) survival curve. Other 63 patients were used for validation cohort. The novel model composed of histologic subtypes, CD19^+^ B cells, natural killer (NK) cells, regulatory T cells, red blood cells (RBC), AST/ALT ratio (SLR), apolipoprotein B (Apo B), and lactic dehydrogenase (LDH). The C-index of this model was 0.784 in the training cohort and 0.735 in the validation cohort. K–M survival curve showed patients with high-risk scores had shorter PFS compared to the low-risk groups. For predicting immune therapy responses, the receiver operating characteristic (ROC), decision curve analysis (DCA), net reclassifcation improvement index (NRI) and integrated discrimination improvement index (IDI) of this model showed better predictive ability compared to EBV DNA. In this study, we constructed a novel model for prognostic prediction and immunotherapeutic response prediction in NPC patients, which may provide clinical assistance in selecting those patients who are likely to gain long-lasting clinical benefits to anti-PD-1 therapy.

**Supplementary Information:**

The online version contains supplementary material available at 10.1007/s00262-023-03626-w.

## Introduction

Nasopharyngeal carcinoma (NPC) has a unique geographical distribution, which occurs more frequently in Southern China, the Middle East and Southeast Asia [1]. Although NPC is a chemosensitive tumor, refractory recurrence and/or metastatic nasopharyngeal carcinoma (R/M NPC) patients with first-line chemotherapy have a poor prognosis [2], with a median overall survival (OS) of 15.7 months [3]. Epstein–Barr virus (EBV) infection plays an important role in the occurrence and development of NPC [4], and the common feature of EBV-positive NPCs is the dense lymphocyte infiltration in the tumor stroma and programmed death ligand-1 (PD-L1) overexpression in tumor cells, making it a potential target for immunotherapy, especially immune checkpoint inhibitors (ICIs), such as programmed death-1/programmed death ligand-1 (PD-1/PD-L1) blockade [5–7].

The ICIs-based therapy has made a breakthrough and was approved for the treatment of refractory R/M NPC in 2021 in China [8]. Inspired by such a huge success, many clinical trials of ICIs alone or plus chemotherapy have been initiated in R/M NPC patients. Based on the result of CAPTAIN-1 [9], the median progression-free survival (PFS) in camrelizumab group (9.7 months) was significantly longer than in the placebo group (6.9 months). Based on the result of JUPITER-02 [10], a significant improvement in median PFS was shown in toripalimab arm (11.7 months) when compared with placebo arm (8.0 months). However, only a small subset of R/M NPC patients can obtain a long-lasting clinical benefits and biomarkers, especially peripheral blood biomarkers, to guide anti-PD-1 treatment choices are lacking, making it urgent to identify reliable predictive peripheral blood biomarkers for prognostic assessment and immunotherapy response prediction in NPC patients.

With advances in high-throughput multiplex assays, various peripheral blood-based immune predictive biomarkers are being identified, such as exosomes [11], cytokines [12, 13], circulating tumor cells and DNA [14, 15]. Xing et al. [16] demonstrated changes in serum PD-L1^+^ tumor extracellular vesicles (TEVs) levels could be a potential predictive biomarker for responses of ICI-based therapy in NPC patients. Peripheral blood testing has the advantage of being minimally invasive and accessible to repeat samples and is widely used in clinical practice. Recently, plasma EBV DNA has been established as an effective marker for NPC diagnostic and prognostic monitoring [17, 18]. A study (POLARIS-02) [19] evaluated the efficacy of toripalimab in R/M NPC and showed patients with a decrease of 50% or more in plasma EBV DNA titer at day 28 had a remarkably better objective response rate (ORR) than those with a decrease of less than 50%. Wang et al. and Xu et al. [20] suggested that longitudinal plasma EBV DNA dynamics monitoring could be used as a predictive biomarker in predicting long-term outcomes in R/M NPC patients receiving immunotherapy. However, the baseline EBV DNA has a poor predictive value for immunotherapy of NPC patients. Therefore, the development of effective model for NPC immunotherapy prediction is of paramount importance.

It has become abundantly clear that the presence, activation, and stimulation of all lymphoid components of the immune system are critical for a successful antitumor immune response, including CD8^+^ T cells, CD4^+^ T cells, B cells, natural killer (NK) cells, and so on [21]. Besides, it has been reported that the response of ICIs in cancer patients is related to the quality and intensity of T cell, NK cell, and B cell responses in the tumor microenvironment [22] and in peripheral blood [23]. Diehn et al. [24] demonstrated that fewer circulating CD8 T cells before immunotherapy was significantly associated with durable clinical benefit in non-small cell lung cancer (NSCLC). However, a correlation between peripheral lymphocytes and immunotherapy response in NPC has rarely been reported.

To the best of our knowledge, there have been few reports on the construction of models that combine absolute counts of lymphocyte subpopulations with other peripheral blood indicators for prognostic assessment and clinical response prediction in R/M NPC patients. In this study, we introduce baseline absolute counts of lymphocyte subpopulations in peripheral blood and other blood indicators to assess their association with prognostic assessment and efficacy prediction in R/M NPC patients. We construct a predictive model that is able to predict the prognostic and immunotherapy response in R/M NPC patients based on their baseline peripheral outcomes, which may provide clinical assistance in selecting those patients who are likely to achieve long-lasting clinical benefits from ICIs-based therapy.

## Methods

### Patients and data collection

This retrospective study enrolled 193 patients and was conducted at Sun Yat-sen University Cancer Center from May 2018 to May 2022, and the deadline for follow-up was January 2023. For enrolled patients, the inclusion/exclusion criteria were as follows: (1) patients had a pathologic confirmation of nasopharyngeal diagnosis; (2) patients must be identified as stage III/IV and diagnosed with recurrent and/or metastatic NPC; (3) patients received anti-PD-1 therapy (toripalimab, camrelizumab, sintilimab or pembrolizumab) and/or plus chemotherapy or radiotherapy; (4) patients were followed up with radiographic tumor evaluation every 1–2 months; (5) lymphocyte subpopulations, biochemical indexes, and blood routine test were measured within a week before the first immunotherapy; (6) patients who lacked any of the blood examination or who had lost to follow-up should be excluded.

In this study, patients were randomly divided into training cohort and validation cohort, and we used the training cohort to construct the predictive model and validate the model in the validation cohort. Patients were defined using response criteria in solid tumors (RECIST) 1.1. based on computed tomography (CT) or magnetic resonance imaging (MRI) results, their responses to anti-PD-1 treatment were evaluated as complete response (CR), partial response (PR), stable disease (SD), or progressive disease (PD).

All experimental data and clinical information of patients were obtained from the electronic medical record system. We collected clinical characteristics (gender, age, ECOG, TNM stage, histological type, clinical stage, treatment and outcomes), blood routine test data, plasma EBV DNA copy number, and biochemical indexes of patients. Lymphocytes are presumed to be key cells in anti-tumor immunity and play a critical role in immunotherapy, therefore, lymphocyte subsets (CD19^+^, CD3^+^, CD3CD4^+^, CD3CD8^+^, CD3^−^CD16^+^CD56^+^, CD4CD25^+^, CD4^+^/CD8^+^, CD8CD25^+^) were detected by flow cytometry in this study.

### Statistical analysis

To develop the prediction model, we employed Lasso Cox regression on the training group to identify markers. Data that were incomplete were excluded, while comprehensive datasets were included in the study. By adjusting the regulation weight *λ*, Lasso performs shrinkage on all regression coefficients toward zero and eliminates the coefficients of many irrelevant features by setting them to zero. The formula used for the prediction model is presented below: risk score = ${\sum }_{{\varvec{i}}}^{{\varvec{n}}}{{\varvec{X}}}_{{\varvec{i}}}\times {{\varvec{Y}}}_{{\varvec{i}}}$ (*n*: number of the inclusion marker, *X*: coefficients, *Y*: survival-related index). Afterward, we compared the performance of the novel prediction model with TNM stage, treatment, and EBV DNA by using the Harrell concordance index, receiver operating characteristic (ROC) curves and decision curve analysis. We utilized the “nomogram” function from the R package to develop a nomogram for predicting the 6-month, 1-year, and 2-year survival rates of NPC patients. After comparing the actual survival rate with the predicted probability of survival, calibration curves were used to calibrate the nomogram for predicting 6-month, 1-year, and 2-year survival rates. NPC patients were classified into low-risk and high-risk groups according to the risk score’s optimal cutoff (“survminer” R package). The Kaplan–Meier method and log-rank test were used to compare the OS of two risk groups. A box plot was used to display the variations in each prognostic index signature between the high-risk and low-risk groups. Sankey diagrams were created to display how patients moved between prognostic risk scores, disease control and survival status. Throughout all statistical analyses, variables with a *P* value less than 0.05 were considered to be statistically significant. Statistical analysis was conducted using R software (version 4.2.1.).

## Result

### Patients’ characteristics

A total of 193 patients (143 men [74.1%]; 50 women [25.9%]; median age 47 years [range 19–75 years]) treated with anti-PD-1 antibody (sintilimab, tislelizumab, toripalimab, or camrelizumab) from Sun Yat-sen University Cancer Center between 2018 and 2022 were included in this study, and detailed patient clinical characteristics and clinicopathological variables in the training (*n* = 130) and validation (*n* = 63) cohorts are listed in Table S1. In this study, all patients were in the advanced stage (stage III 59 [30.6%]; stage IV 134 [69.4%]) and underwent recurrence and/or metastasis when treated with anti-PD-1 antibody. In addition, pseudoprogression typically occurred within the first few weeks of ICI-based treatment, so the response to immunotherapy in NPC patients was initially assessed at 4–6 months and continuously updated. Statistical results indicated that 38 (29.2%) patients in the training cohort and 17 (27.0%) patients in the validation cohort showed partial response. The number of stable diseases in the training and validation cohorts is 70 (53.8%) and 36 (57.1%), respectively, and 32 (training cohort 22 [16.9%]; validation cohort 10 [15.9%]) patients developed progressive disease.

### Construction and evaluation of prediction model

**Fig. 1:**
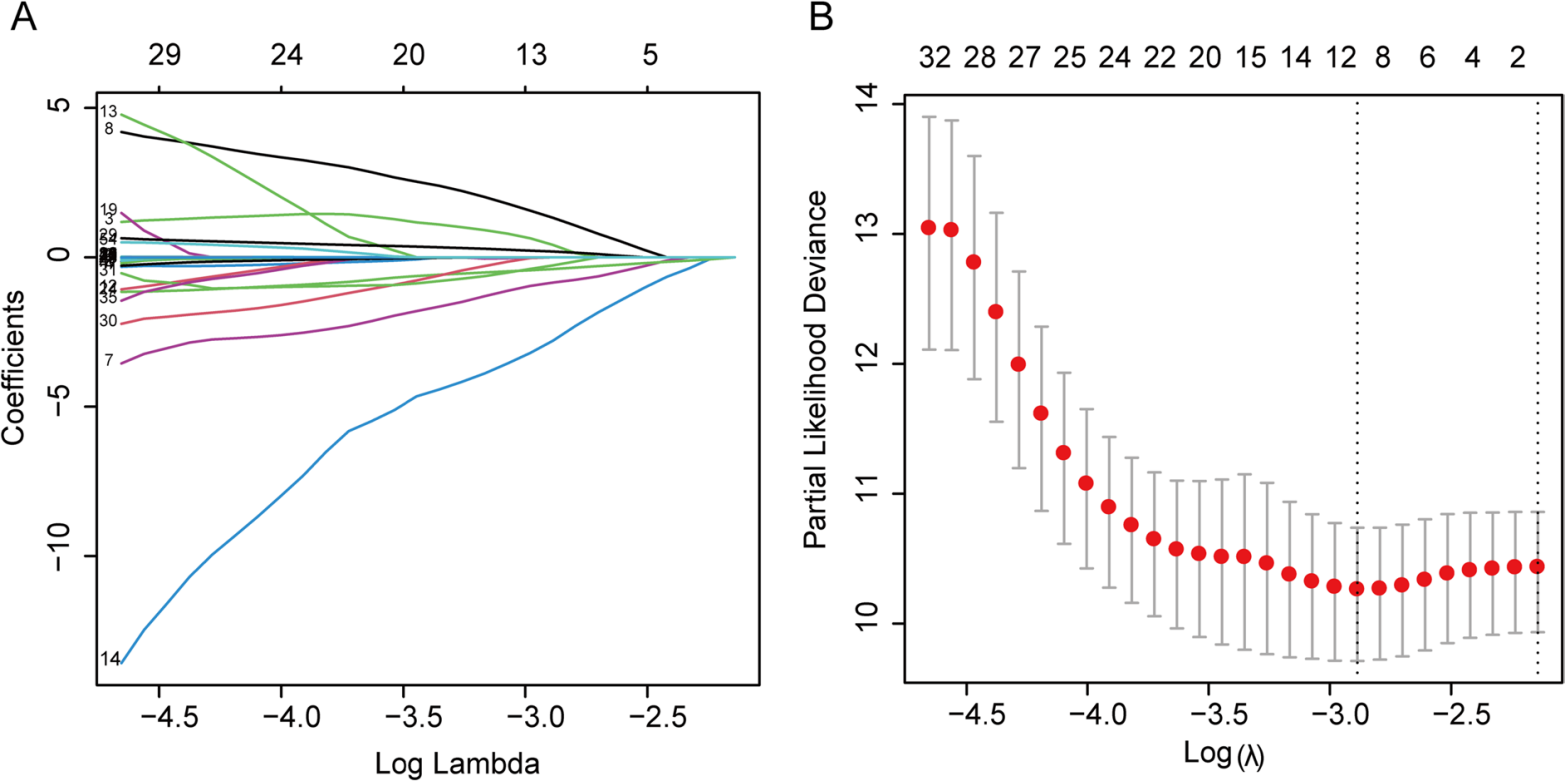
Construction of a prediction model based on the peripheral blood biomarkers. **A** Prognosis model construction in the development cohort by Lasso Cox regression analysis. **B** Cross-validation of tuning parameter selection in the Lasso Cox regression

In the training cohort, we used Lasso Cox regression analysis to extract the most relevant predictor variables and constructed an 8-prognostic index signature (histological type, CD19^+^ B cells, NK cells, CD8^+^CD25^+^ regulatory T cells [Treg], red blood cells [RBC], aspartate aminotransferase [AST]/alanine aminotransferase [ALT] ratio [SLR], apolipoprotein B [Apo B], and lactic dehydrogenase [LDH]) from 57 baseline levels of peripheral blood-based biomarkers. The coefficient profile plot and cross-validation for tuning the parameter selection are shown in Fig. 1A, B. We develop a formula for the disease progression of each patient and calculate the prediction model as follows: risk score = (0.4144*histological type) + (− 0.8462*CD19^+^ B cells) + (1.3227*NK cells) + (− 2.7933*CD8^+^ CD25^+^ Treg cells) + (− 0.3935*RBC) + (0.1929*ALT/AST) + (− 0.2713*Apo B) + (0.0015*LDH).

**Fig. 2:**
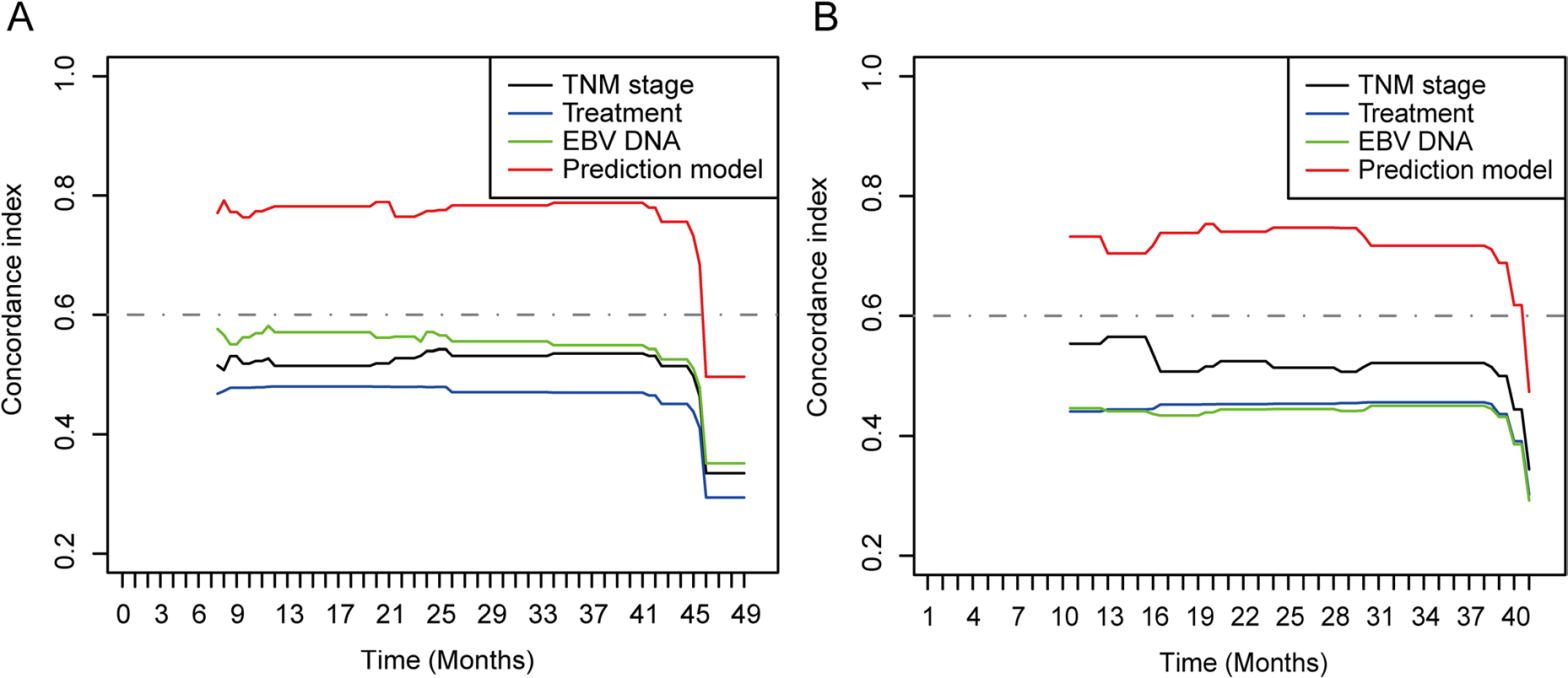
The C-index of PFS for prediction model, TNM stage, treatment, and EBV in the training cohort (**A**) and validation cohort (**B**)

We used the concordance index (C-index) to compare the prognostic prediction power of this model with that of EBV DNA levels, TNM stage, and treatment. As shown in Table 1 and Fig. 2A, B, the C-index of this prediction model for PFS was 0.784 (95% CI 0.714–0.853), which was the highest among the TNM stage (0.545, 95% CI 0.466–0.624), treatment (0.521, 95% CI 0.451–0.591) and EBV DNA (0.560, 95% CI 0.485–0.675) in training cohort (*P* < 0.0001). Similar results could be observed in the validation patient cohort, and this prediction model had the highest C-index among the TNM stage (0.555, 95% CI 0.452–0.657), treatment (0.509, 95% CI 0.399–0.620) and EBV DNA (0.519, 95% CI 0.389–0.650).

**Table 1:** The C-index of PFS for prediction model, TNM stage, treatment, and EBV DNA

Survival prediction	C-index	95 CI%	*P*
*For training cohort*			
Prediction model	0.784	0.714–0.853	
TNM stage	0.545	0.466–0.624	
Treatment	0.521	0.451–0.591	
EBV DNA	0.560	0.485–0.675	
Prediction model versus TNM stage			< 0.001
Prediction model versus treatment			< 0.001
Prediction model versus EBV DNA			< 0.001
*For validation cohort*			
prediction model	0.735	0.616–0.853	
TNM stage	0.555	0.452–0.657	
Treatment	0.509	0.399–0.620	
EBV DNA	0.519	0.389–0.650	
Prediction model versus TNM stage			0.002
Prediction model versus treatment			0.015
Prediction model versus EBV DNA			0.013

C-index = concordance index; *P* values are calculated based on normal approximation using function rcorrp.cens in Hmisc package

### Nomogram development with a risk score, TNM stage, treatment, and EBV-DNA

**Fig. 3:**
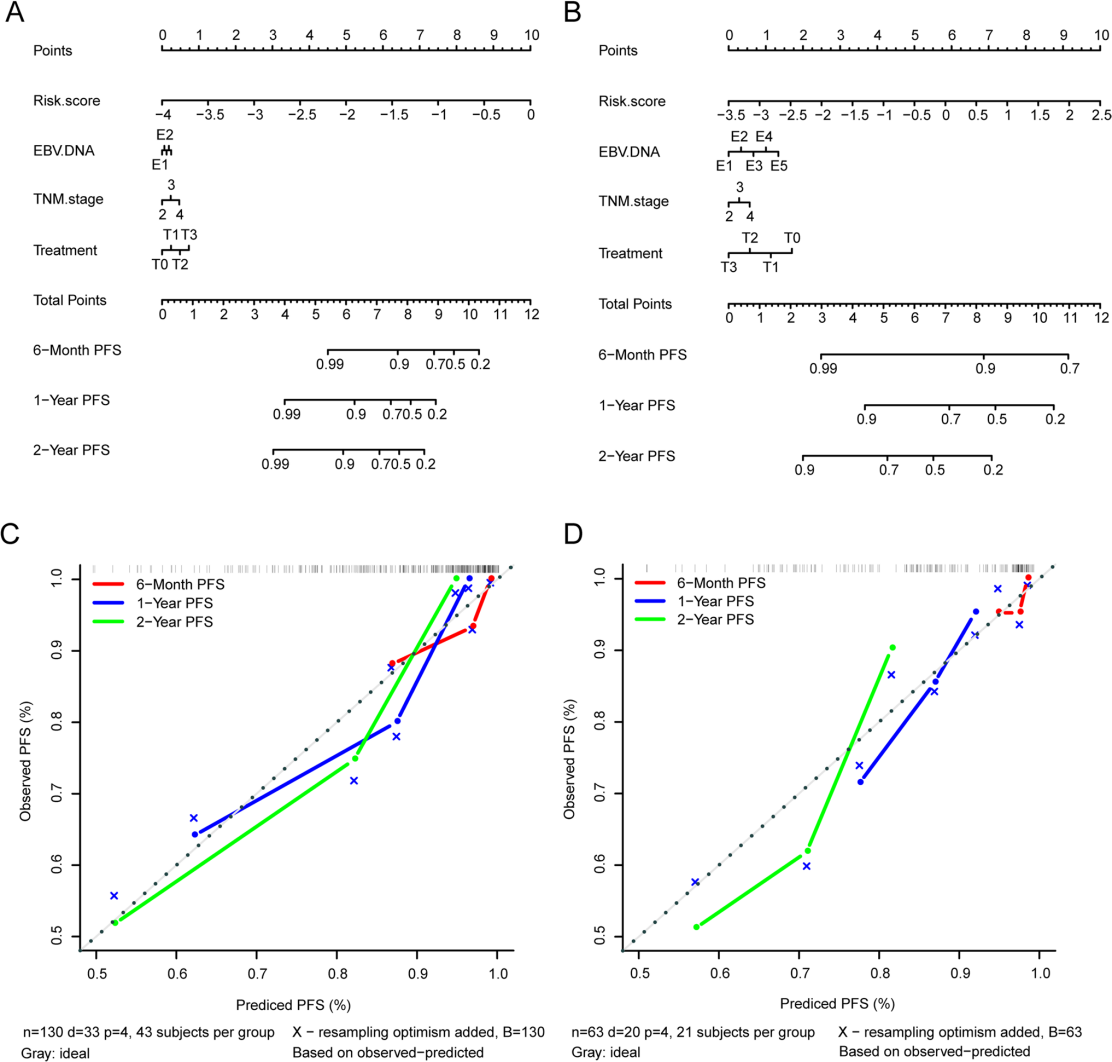
Efficacy prediction of immunotherapy in NPC patients based on risk score and EBV DNA. **A**, **B** Nomogram predicting the six-month, one- and two-year PFS in the training cohort (**A**) and validation cohort (**B**). **C**, **D** Calibration curves of nomogram for PFS in the training cohort (**C**) and validation cohort (**D**). T0, radiotherapy plus anti-PD-1 treatment; T1, chemotherapy plus anti-PD-1 treatment; T2, radiotherapy and chemotherapy plus anti-PD-1 treatment; T3, only anti-PD-1 treatment. E1, EBV DNA < 10^3^; E2, EBV DNA 10^3^–10^4^; E3, EBV DNA 10^4^–10^5^; E4, EBV DNA 10^5^–10^6^; E5, EBV DNA > 10.^6^

Based on the prognostic risk score, EBV DNA levels, TNM stage, and treatment, we constructed a nomogram for the prognostic prediction in the training (Fig. 3A) and validation (Fig. 3B) cohort. This nomogram allows users to predict the probability of 6-month, 1- and 2-year PFS according to the combination of covariates for a patient. For example, the patient’s risk score is found, and a straight line is drawn upward to the “Points” axis to confirm the score. Repeat this process for each variable, and sum the scores, then place this sum on the “Total Points” axis, and find the corresponding PFS to predict the likelihood of six-month, one- and two-year PFS. The calibration curves of 6-month 1-year, and 2-year survival showed ideal consistency between the established nomogram and the actual observations in training (Fig. 3C) and validation cohort (Fig. 3D).

### Risk stratifcation of PFS based on the prediction model

**Fig. 4:**
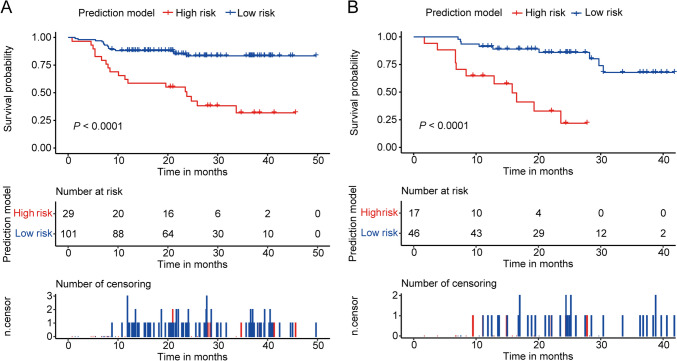
Kaplan–Meier survival curves for 4-year PFS of R/M NPC patients in the high-risk group and low-risk group in the training (**A**) and validation cohorts (**B**)

Based on the optimal cutoff value of the risk score, the patients were subdivided into a high-risk (*n* = 46) or low-risk (*n* = 147) group. The Kaplan–Meier survival curves showed that patients with low risk had a longer PFS than those with high risk in the training cohort and validation cohort (*P* < 0.0001, Fig. 4A, B). In addition, we analyzed the differences in the number of RBC, B cells, NK cells, Treg cells, apo B, LDH, LSR and histological type between the high-risk and low-risk groups. The number of RBC (training cohort: *P* < 0.001, validation cohort: *P* < 0.001), B cells (training cohort: *P* = 0.002, validation cohort: *P* = 0.011) and Treg cells (training cohort: *P* < 0.001, validation cohort: *P* = 0.042) in high-risk group were significantly higher than those in low-risk group (Table S2).

### The performance of the prediction model in predicting immunotherapy response

**Fig. 5:**
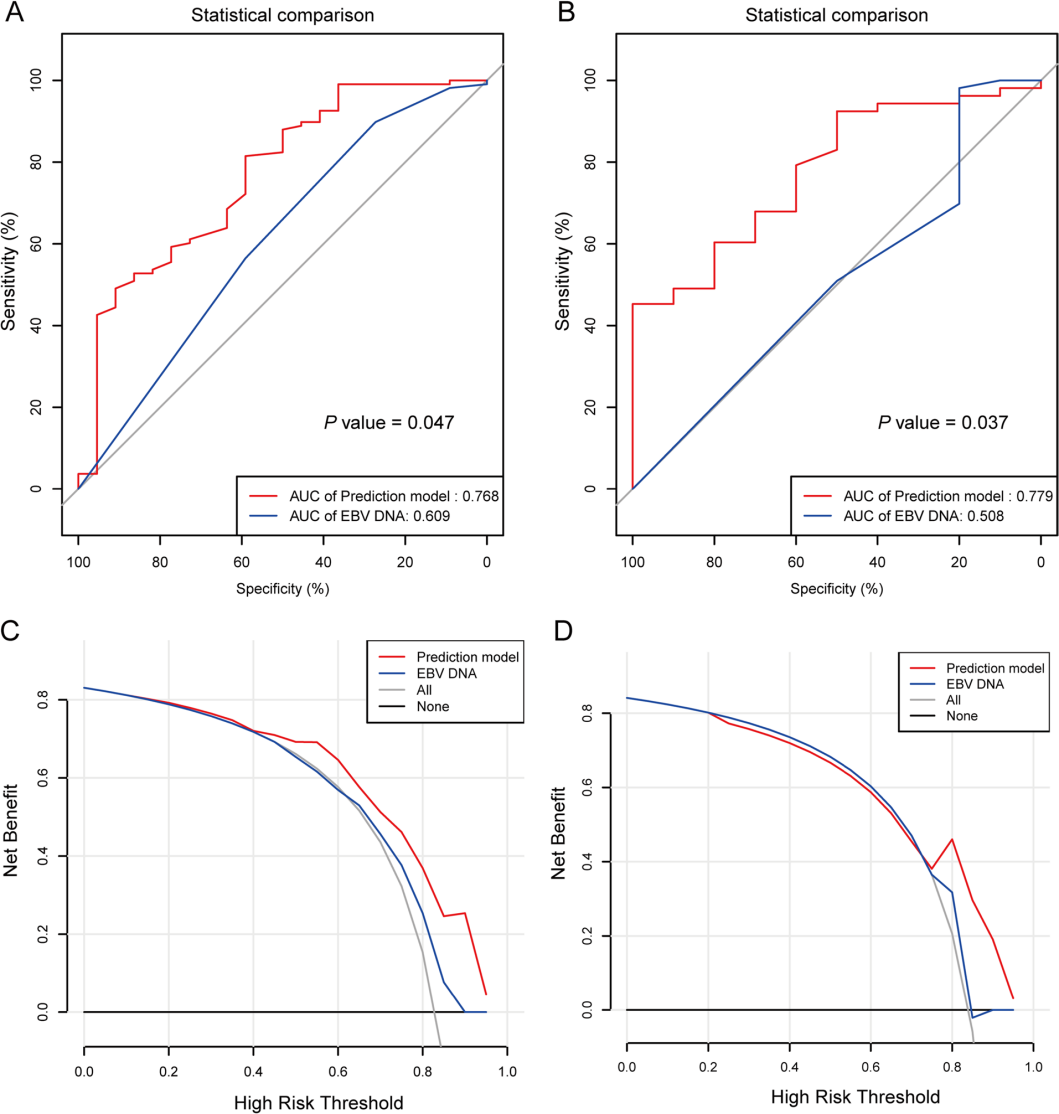
The prediction accuracy of the prediction model efficacy in R/M NPC patients. ROC curves of the prediction model and EBV DNA in the training (**A**) and validation cohorts (**B**). Decision curve analysis for the prediction model compared with EBV DNA in the training (**C**) and validation cohorts (**D**). The horizontal black line represents the net benefit when all NPC patients are considered to have no outcome

As shown in Table 2, patients were divided into low-risk (training cohort: *n* = 101, validation cohort: *n* = 46) and high-risk (training cohort: *n* = 101, validation cohort: *n* = 46) based on the optimal cutoff value of risk scores. In order to evaluate the accuracy of this prediction model, we analyzed the ROC and compared the area under the curve (AUC) with EBV DNA that was an effective predictive biomarker. As shown in Fig. 5, the prediction model had an AUC of 0.768 [95% CI 0.686–0.837, standard error (SE) 0.066], which was higher than EBV DNA (AUC: 0.609, SE 0.058, 95% CI 0.519–0.693, *P* = 0.047) in training cohort (Fig. 5A). A similar predictive accuracy of the prediction model was obtained in the validation cohort (Fig. 5B) (AUC: 0.779, SE 0.074, 95% CI 0.657–0.874), which was higher than EBV DNA (AUC: 0.508, SE 0.102, 95% CI 0.378–0.636, *P* = 0.037).

**Table 2:** Response to immunotherapy based on the prediction model

Survival prediction	Low	High	*χ*2	*P*
*For training cohort (n = 130)*	*n* = 101	*n* = 29		
Best overall response-no. (%)			12.134	0.002
Partial response (PR)	33 (32.7)	5 (17.2)		
Stable disease (SD)	57 (56.4)	13 (44.8)		
Progressive disease (PD)	11 (10.9)	11 (37.9)		
*For validation cohort (n = 63)*	*n* = 46	*n* = 17		
Best overall response-no. (%)			7.603	0.022
Partial response (PR)	15 (32.6)	2 (11.8)		
Stable disease (SD)	27 (58.7)	9 (52.9)		
Progressive disease (PD)	4 (8.7)	6 (35.3)

**Fig. 6:**
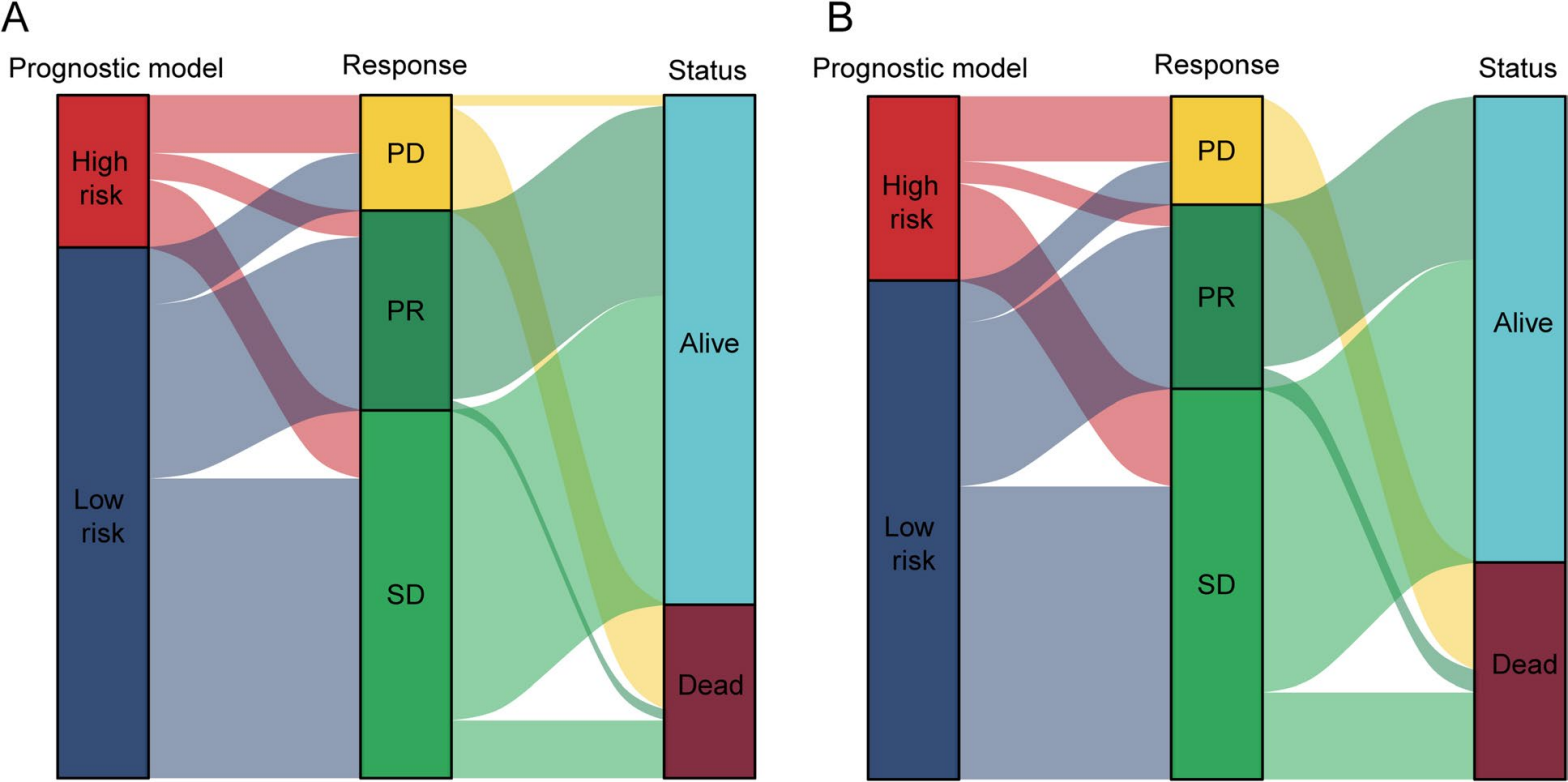
NPC patients transferred between the risk score, response and survival status in the training (**A**) and validation cohort (**B**)

In addition, the decision curve analysis (DCA) showed that prediction model curve was higher than the EBV DNA curve in the training (Fig. 5C) and validation cohort (Fig. 5D), which suggested the superior predictive effects in this prediction model compared with EBV DNA. Furthermore, we analyzed net reclassification improvement (NRI) and integrated discrimination improvement (IDI) to assess reclassification performance and improvement in discrimination of prediction model. As shown in Table 3, the NRI and IDI had a great improvement in training (NRI% 78.79, *P* < 0.001; IDI% 16.06, *P* = 0.003) and validation cohort (NRI% 18.11, *P* = 0.593; IDI% 3.88, *P* = 0.113). These results demonstrated this prediction model had a superior potential predictive performance compared with EBV DNA. Moreover, Sankey diagrams showed that most of the high-risk group NPC patients shifted to PD group and had a lower level of survival status in the training (Fig. 6A) and validation cohort (Fig. 6B).

**Table 3:** The NRI and IDI were used to assess reclassification performance and improvement in discrimination of prediction model

	NRI%	*P* value	IDI%	*P* value
*Training cohort*				
Prediction model versus EBV DNA	78.79	< 0.001	16.06	0.003
*Validation cohort*				
Prediction model versus EBV DNA	18.11	0.593	3.88	0.113

NRI, net reclassification improvement index; IDI, integrated discrimination improvement index

## Discussion

Although ICI-based therapies, primarily represented by PD-1/PD-L1 inhibitors, significantly improve the overall survival in R/M NPC patients, only a few of NPC patients can achieve long-term benefits. For ICI-based therapies to provide maximum benefit to NPC patients, a number of markers that predict the efficacy of immunotherapy have been identified over the years, including tumor mutation burden (TMB) [25, 26], tumor infiltrating lymphocytes (TILs) [27] detection, and PD-L1 expression in tumor tissue [28, 29]. However, these biomarkers are difficult to popularize in clinical practice for their inherent limitations. For PD-L1 expression, there is spatiotemporal heterogeneity and its expression is different in different biopsy sites. As for TMB, the gold detection standard is whole exome sequencing (WES) [30] which is expensive and has a long detection cycle [31]. In addition, the efficacy of TILs as predictive markers for ICI-based therapies needs further exploration and validation by more clinical trials. Peripheral blood biomarkers, which have the advantage of being minimally invasive and potentially repeatable and sequentially monitored, have been shown to be a potential tool for predicting immunotherapy response.

In this study, based on baseline peripheral blood lymphocyte subpopulations, biochemical indexes and blood routine tests, we utilize Lasso Cox regression to select eight predictive indicators [histologic subtypes, CD19^+^ B cells, NK cells, regulatory T cells, RBC, AST/ALT ratio (SLR), Apo B, and LDH] for disease progression prediction of R/M NPC patients treated with anti-PD-1 antibody. We demonstrate that this prediction model is associated with response to anti-PD-1 therapy in R/M NPC patients. Moreover, this model is able to predict the progression-free survival in R/M NPC patients.

Nine kinds of immune cell subpopulations of peripheral blood (CD19^+^, CD3^+^, CD3^−^CD16^+^CD56^+^, CD3^+^CD4^+^, CD3^+^CD8^+^, CD4^+^/CD8^+^, CD4^+^CD25^+^, CD8^+^CD25^+^, CD3CD19CD56) were selected for the development of this prediction model. Treg (CD8^+^CD25^+^) cells are able to inhibit some immune cells activity and suppress effective anti-tumor immunity. Therefore, strategies to inhibit or deplete Tregs are being explored in cancer immunotherapy [32–34]. Some studies [35] have shown that Treg cells are infiltrated into tumor tissues, which is often correlated with poor prognosis [36]. NK cells are innate lymphocyte population with unique ability to rapidly kill infected, transformed, allogeneic, or stressed cells without any prior encounter [37, 38]. Substantial evidence [39–41] supports a crucial role for NK cells in predicting anti-PD-1 efficacy and routine surveillance against cancer. CD19^+^ B cells are involved in a variety of immune responses and their dysregulation can lead to immune system disorders [42, 43]. They are also a target of certain therapies [44, 45], such as CAR-T cell therapy for B cell malignancies, where the patient’s own T cells are genetically engineered to recognize and kill CD19^+^ B cells. Besides, a series of studies have found that the number of CD8^+^ T cells or the ratio of CD8^+^/CD4^+^ T cells in the tumor microenvironment are correlated with ICIs outcomes and serve as positive predictors of immunotherapy [46, 47]. Huang et al. [48] brought T lymphocytes subpopulations into studies and identified that CD3^−^CD16^+^CD56^+^ cells, CD3^+^CD4^+^ cells and Treg cells were significantly correlated with the prognostic of NSCLC patients. Chi et al. [49] constructed a prognostic risk score that includes the population of immune cells and was able to provide accurate prediction of the response in patients with malignant melanoma treated with ICI therapy.

LDH is released into the blood when cells are damaged or ruptured. Therefore, the level of LDH in the blood is commonly used to assess the extent of tissue damage [50]. LDH is considered an important and potential biomarker in cancer immunotherapy [51–53]. LDH level and SLR were identified to be reliable prognostic biomarkers in NPC patients by a number of studies. Zhang et al. [54] confirmed that dynamic changes of LDH and LSR were correlated with the prognosis of R/M NPC. Li et al. [55] identified that baseline high levels of serum LDH and ALP were adverse prognostic indicators for NPC patients.

In this study, we constructed a prediction model based on these eight factors and calculated the risk score for the disease progression of each R/M NPC patient. This prediction model is not only able to predict prognosis, but also is a useful tool for predicting responses of ICI-based therapies in R/M NPC patients. In addition, our prediction model outperforms TNM stage, treatment, and EBV DNA in terms of prognostic efficacy and provides additional prognostic value to these existing predictors. Moreover, this predictive model with a higher predictive power than EBV DNA. To the best of our knowledge, our study is the first to construct a prediction model index based on baseline absolute counts of lymphocyte subpopulations to predict the PFS and immunotherapy responses in R/M NPC patients treated with ICI-based therapies. Our findings demonstrate the utility of peripheral blood indicators as a predictive tool for identifying R/M NPC patients more likely to respond to PD-1 antibodies, which could help identify responders and non-responders early and avoid unnecessarily prolonged treatment.

However, several limitations in our study should be noted. First, this retrospective study was based on a single center, which may lead to unavoidable selection bias. Therefore, in future research, we plan to make greater efforts to expand the sample size and engage in collaborations with multiple hospitals to conduct multicenter studies to further validate this model. Second, we did not clarify the molecular mechanisms and associations between these eight indicators and the immune microenvironment.

## Conclusion

In summary, we proposed a novel prognostic nomogram model based on peripheral blood biomarkers and emphasized their importance as potential prognostic biomarkers for the treatment of NPC with anti-PD-1/PD-L1. As our proposed model is valuable tool for assessing NPC patient’s eligibility for anti-PD-1 therapy, further investigations are needed to evaluate the predictive value of these markers in larger multicenter populations and prospective clinical studies.

### Supplementary Information

Below is the link to the electronic supplementary material.

Supplementary file1 (PDF 191 KB)

## Data Availability

The datasets used or analyzed during the current study are available from the corresponding author on reasonable request.

## References

[1] Chang ET (2021). The evolving epidemiology of nasopharyngeal carcinoma. Cancer Epidemiol Biomark Prev.

[2] Chen YP (2019). Nasopharyngeal carcinoma. Lancet.

[3] Wang WY (2013). Long-term survival analysis of nasopharyngeal carcinoma by plasma Epstein–Barr virus DNA levels. Cancer.

[4] Tsang CM (2014). Epstein–Barr virus infection and persistence in nasopharyngeal epithelial cells. Chin J Cancer.

[5] Huang D (2017). Epstein–Barr virus-induced VEGF and GM-CSF drive nasopharyngeal carcinoma metastasis via recruitment and activation of macrophages. Cancer Res.

[6] Pathmanathan R (1995). Clonal proliferations of cells infected with Epstein–Barr virus in preinvasive lesions related to nasopharyngeal carcinoma. N Engl J Med.

[7] Zhu Q (2017). Tumor cells PD-L1 expression as a favorable prognosis factor in nasopharyngeal carcinoma patients with pre-existing intratumor-infiltrating lymphocytes. Oncoimmunology.

[8] Xu JY (2022). Current status and advances of immunotherapy in nasopharyngeal carcinoma. Ther Adv Med Oncol.

[9] Yang Y (2021). Efficacy, safety, and biomarker analysis of camrelizumab in previously treated recurrent or metastatic nasopharyngeal carcinoma (CAPTAIN study). J Immunother Cancer.

[10] Mai HQ (2021). Toripalimab or placebo plus chemotherapy as first-line treatment in advanced nasopharyngeal carcinoma: a multicenter randomized phase 3 trial. Nat Med.

[11] Chen G (2018). Exosomal PD-L1 contributes to immunosuppression and is associated with anti-PD-1 response. Nature.

[12] Sanmamed MF (2017). Changes in serum interleukin-8 (IL-8) levels reflect and predict response to anti-PD-1 treatment in melanoma and non-small-cell lung cancer patients. Ann Oncol.

[13] Keegan A (2020). Plasma IL-6 changes correlate to PD-1 inhibitor responses in NSCLC. J Immunother Cancer.

[14] Raja R (2018). Early reduction in ctDNA predicts survival in patients with lung and bladder cancer treated with durvalumab. Clin Cancer Res.

[15] Tamminga M (2019). Circulating tumor cells in advanced non-small cell lung cancer patients are associated with worse tumor response to checkpoint inhibitors. J Immunother Cancer.

[16] Xing S (2020). An ultrasensitive hybridization chain reaction-amplified CRISPR-Cas12a aptasensor for extracellular vesicle surface protein quantification. Theranostics.

[17] Twu CW (2007). Comparison of the prognostic impact of serum anti-EBV antibody and plasma EBV DNA assays in nasopharyngeal carcinoma. Int J Radiat Oncol Biol Phys.

[18] Wang WY (2010). Plasma EBV DNA clearance rate as a novel prognostic marker for metastatic/recurrent nasopharyngeal carcinoma. Clin Cancer Res.

[19] Wang FH (2021). Efficacy, safety, and correlative biomarkers of toripalimab in previously treated recurrent or metastatic nasopharyngeal carcinoma: a phase II clinical trial (POLARIS-02). J Clin Oncol.

[20] Xu JY (2022). Association of plasma Epstein–Barr virus DNA with outcomes for patients with recurrent or metastatic nasopharyngeal carcinoma receiving anti-programmed cell death 1 immunotherapy. JAMA Netw Open.

[21] Paijens ST (2021). Tumor-infiltrating lymphocytes in the immunotherapy era. Cell Mol Immunol.

[22] Sade-Feldman M (2018). Defining T cell states associated with response to checkpoint immunotherapy in melanoma. Cell.

[23] An HJ, Chon HJ, Kim C (2021). Peripheral blood-based biomarkers for immune checkpoint inhibitors. Int J Mol Sci.

[24] Nabet BY (2020). Noninvasive early identification of therapeutic benefit from immune checkpoint inhibition. Cell.

[25] Barroso-Sousa R (2020). Prevalence and mutational determinants of high tumor mutation burden in breast cancer. Ann Oncol.

[26] Marabelle A (2020). Association of tumour mutational burden with outcomes in patients with advanced solid tumours treated with pembrolizumab: prospective biomarker analysis of the multicohort, open-label, phase 2 KEYNOTE-158 study. Lancet Oncol.

[27] Gataa I (2021). Tumour-infiltrating lymphocyte density is associated with favourable outcome in patients with advanced non-small cell lung cancer treated with immunotherapy. Eur J Cancer.

[28] Chen S (2019). Mechanisms regulating PD-L1 expression on tumor and immune cells. J Immunother Cancer.

[29] Doroshow DB (2021). PD-L1 as a biomarker of response to immune-checkpoint inhibitors. Nat Rev Clin Oncol.

[30] Tarazona N (2019). Targeted next-generation sequencing of circulating-tumor DNA for tracking minimal residual disease in localized colon cancer. Ann Oncol.

[31] Jardim DL (2021). The Challenges of tumor mutational burden as an immunotherapy biomarker. Cancer Cell.

[32] Tay C, Tanaka A, Sakaguchi S (2023). Tumor-infiltrating regulatory T cells as targets of cancer immunotherapy. Cancer Cell.

[33] Van Damme H (2021). Therapeutic depletion of CCR8^+^ tumor-infiltrating regulatory T cells elicits antitumor immunity and synergizes with anti-PD-1 therapy. J Immunother Cancer.

[34] Lee JC (2020). Regulatory T cell control of systemic immunity and immunotherapy response in liver metastasis. Sci Immunol.

[35] Tanaka A, Sakaguchi S (2019). Targeting Treg cells in cancer immunotherapy. Eur J Immunol.

[36] Suzuki S (2020). Immune-checkpoint molecules on regulatory T-cells as a potential therapeutic target in head and neck squamous cell cancers. Cancer Sci.

[37] Liu S (2021). NK cell-based cancer immunotherapy: from basic biology to clinical development. J Hematol Oncol.

[38] Myers JA, Miller JS (2021). Exploring the NK cell platform for cancer immunotherapy. Nat Rev Clin Oncol.

[39] Shimasaki N, Jain A, Campana D (2020). NK cells for cancer immunotherapy. Nat Rev Drug Discov.

[40] Laskowski TJ, Biederstädt A, Rezvani K (2022). Natural killer cells in antitumour adoptive cell immunotherapy. Nat Rev Cancer.

[41] Subrahmanyam PB (2018). Distinct predictive biomarker candidates for response to anti-CTLA-4 and anti-PD-1 immunotherapy in melanoma patients. J Immunother Cancer.

[42] Wang SS (2019). Tumor-infiltrating B cells: their role and application in anti-tumor immunity in lung cancer. Cell Mol Immunol.

[43] Kim SS (2021). Role of B cells in responses to checkpoint blockade immunotherapy and overall survival of cancer patients. Clin Cancer Res.

[44] Qi Y (2022). Efficacy and safety of CD19-specific CAR T cell-based therapy in B-cell acute lymphoblastic leukemia patients with CNSL. Blood.

[45] Wang T (2023). Coadministration of CD19 and CD22 directed chimeric antigen receptor T-cell therapy in childhood B-cell acute lymphoblastic leukemia: a single-arm, multicenter. Phase II Trial J Clin Oncol.

[46] Chen X (2021). CD8^+^ T effector and immune checkpoint signatures predict prognosis and responsiveness to immunotherapy in bladder cancer. Oncogene.

[47] Jiang P (2018). Signatures of T cell dysfunction and exclusion predict cancer immunotherapy response. Nat Med.

[48] Huang H (2022). Development and validation of a nomogram for evaluating the prognosis of immunotherapy plus antiangiogenic therapy in non-small cell lung cancer. Cancer Cell Int.

[49] Chi P (2022). An immune risk score predicts progression-free survival of melanoma patients in South China receiving anti-PD-1 inhibitor therapy-a retrospective cohort study examining 66 circulating immune cell subsets. Front Immunol.

[50] Kristjansson RP (2016). Common and rare variants associating with serum levels of creatine kinase and lactate dehydrogenase. Nat Commun.

[51] Wagner NB (2018). S100B and LDH as early prognostic markers for response and overall survival in melanoma patients treated with anti-PD-1 or combined anti-PD-1 plus anti-CTLA-4 antibodies. Br J Cancer.

[52] Peng L (2020). Peripheral blood markers predictive of outcome and immune-related adverse events in advanced non-small cell lung cancer treated with PD-1 inhibitors. Cancer Immunol Immunother.

[53] Capone M (2018). Baseline neutrophil-to-lymphocyte ratio (NLR) and derived NLR could predict overall survival in patients with advanced melanoma treated with nivolumab. J Immunother Cancer.

[54] Zhang A (2021). Dynamic serum biomarkers to predict the efficacy of PD-1 in patients with nasopharyngeal carcinoma. Cancer Cell Int.

[55] Li G (2012). Increased pretreatment levels of serum LDH and ALP as poor prognostic factors for nasopharyngeal carcinoma. Chin J Cancer.

