# Supplementary material for: A novel model for predicting prognosis and response to immunotherapy in nasopharyngeal carcinoma patients
Source: Cancer Immunol Immunother. 2024 Jan 18;73(1):14. doi: 10.1007/s00262-023-03626-w (PMC10796600; doi:10.1007/s00262-023-03626-w)
Supplement: Supplementary file 1 — Supplementary file1 (PDF 191 KB) [file 262_2023_3626_MOESM1_ESM.pdf]

# Supplementary Information

**Table 1** Demographics and clinical characteristics of patients in the development and validation cohort

| Characteristic | Development cohort | Validation cohort |
|---|---|---|
| | **n = 130** | **n = 63** |
| | **No. (%) or Mean±sd** | **No. (%) or Mean±sd** |
| Gender | | |
| Male | 37 (28.5%) | 13 (20.6%) |
| Female | 93 (71.5%) | 50 (79.4%) |
| Age (years) | 47.18 (11.32%) | 46.38 (10.11%) |
| BMI (kg/m$^2$) | 22.46±3.23 | 22.99±3.46 |
| NRI | 109.8±8.70 | 110.55±8.30 |
| TNM stage | | |
| II | 4 (3.1%) | 1 (1.6%) |
| III | 35 (26.9%) | 21 (33.3%) |
| IV | 91 (70.0%) | 41 (65.1%) |
| ECOG | | |
| 0 | 10 (7.7%) | 5 (7.9%) |
| 1 | 117 (90.0%) | 55 (87.3%) |
| 2 | 3 (2.3%) | 3 (4.8%) |
| Histological type | | |
| H0 | 126 (96.6%) | 61 (96.8%) |
| H1 | 4 (3.1%) | 2 (3.2%) |
| Treatment | | |
| T0 | 8 (6.2%) | 10 (15.9%) |
| T1 | 107 (82.3%) | 50 (79.4%) |
| T2 | 13 (10.0%) | 2 (3.2%) |
| T3 | 2 (1.5%) | 1 (1.6%) |
| Type | | |
| 0 | 22 (16.9%) | 10 (15.9%) |
| 1 | 108 (83.1%) | 53 (84.1%) |
| WBC ($10^9$/L) | 7.01±2.47 | 6.22±2.83 |
| Neutrophils ($10^9$/L) | 4.94±2.16 | 4.25±2.44 |
| Lymphocyte ($10^9$/L) | 1.45±0.68 | 1.39±0.61 |
| Monocyte ($10^9$/L) | 0.44±0.19 | 0.42±0.20 |
| Eosinophil ($10^9$/L) | 0.15±0.14 | 0.13±0.10 |
| HGB (g/L) | 136.81±18.60 | 134.67±17.59 |
| MCH (pg) | 29.05±3.38 | 28.95±2.98 |
| MCHC (g/L) | 323.72±14.50 | 324.10±13.52 |
| Platelet ($10^9$/L) | 273.65±80.88 | 267.98±98.82 |
| RBC ($10^{12}$/L) | 4.74±0.64 | 4.71±0.83 |
| B cells ($10^9$/L) | 0.17±0.13 | 0.15±0.11 |

| | | |
|---|---|---|
| NK cells ($10^9$/L) | 0.32±0.20 | 0.33±0.20 |
| T cells ($10^9$/L) | 0.93±0.54 | 0.90±0.50 |
| CD4+cells ($10^9$/L) | 0.45±0.30 | 0.40±0.26 |
| CD8+cells ($10^9$/L) | 0.43±0.28 | 0.42±0.25 |
| CD4CD8+cells ($10^9$/L) | 1.17±0.66 | 1.03±0.50 |
| CD41+cells ($10^9$/L) | 0.24±0.18 | 0.21±0.15 |
| CD81+cells ($10^9$/L) | 0.15±0.10 | 0.15±0.11 |
| ALB (g/L) | 44.79±3.56 | 45.03±3.14 |
| ALP (U/L) | 83.88±51.37 | 87.76±45.06 |
| ALT (U/L) | 25.23±23.41 | 26.54±19.23 |
| AST (U/L) | 22.31±12.91 | 23.52±12.53 |
| LSR | 1.14±0.96 | 1.10±0.51 |
| APOA (g/L) | 1.37±0.24 | 1.35±0.25 |
| APOB (g/L) | 0.96±0.22 | 1.01±0.21 |
| ABR | 1.50±0.46 | 1.39±0.38 |
| CRE (μmol/L) | 68.46±15.97 | 73.02±16.26 |
| CRP (mg/L) | 7.42±17.95 | 10.08±19.09 |
| Cys-C (mg/L) | 0.86±0.17 | 0.89±0.18 |
| GGT (U/L) | 36.72±45.50 | 44.39±40.37 |
| GLOB (g/L) | 31.29±5.47 | 31.85±5.11 |
| AGR | 1.47±0.28 | 1.45±0.27 |
| LDH (U/L) | 203.87±76.10 | 241.95±310.20 |
| HDL (mmol/L) | 1.28±0.32 | 1.24±0.31 |
| LDL (mmol/L) | 3.16±0.83 | 3.33±0.97 |
| SAA (mg/L) | 38.60±122.61 | 53.35±165.07 |
| TG (mmol/L) | 1.37±0.82 | 1.51±0.88 |
| TP (g/L) | 76.04±5.67 | 76.88±5.00 |
| UA (μmol/L) | 352.88±96.57 | 369.51±100.9 |
| CAR | 0.20±0.61 | 0.25±0.50 |
| SIRI | 1.93±1.95 | 1.57±2.01 |
| PLR | 241.96±167.39 | 230.26±134.13 |
| LMR | 3.52±1.59 | 3.96±2.71 |
| NLR | 4.34±4.03 | 3.45±2.13 |
| PNI | 52.02±5.18 | 51.99±4.34 |
| LCR | 1.40±2.55 | 1.17±1.66 |
| LMR | 3.52±1.59 | 3.96±2.71 |
| NLR | 4.34±4.03 | 3.45±2.13 |
| PNI | 52.02±5.18 | 51.99±4.34 |
| LCR | 1.40±2.55 | 1.17±1.66 |
| SCR | 10.54±23.07 | 5.24±4.56 |
| LAR | 2.71±1.14 | 2.78±1.38 |
| ALI | 342.82±209.66 | 387.55±195.62 |
| EBV-DNA | | |
| $<10^3$ | 70(53.8%) | 32(50.8%) |

| | | |
|---|---|---|
| $10^3$-$10^4$ | 43(33.1%) | 13(20.6%) |
| $10^4$-$10^5$ | 13(10.0%) | 15(23.8%) |
| $10^5$-$10^6$ | 3(2.3%) | 2(3.2%) |
| $>10^6$ | 1(0.8%) | 1(1.6%) |

Histological type: H1, poorly differentiated; H0, undifferentiation. Treatment: T0, radiotherapy plus anti-PD-1 treatment; T1, chemotherapy plus anti-PD-1 treatment; T2, radiotherapy and chemotherapy plus anti-PD-1 treatment; T3, only anti-PD-1 treatment.

**Supplementary Table 2**. Predictor variables in high risk group and low risk group

| Predictor variables | High risk group n (%) or mean (95 CI%) | Low risk group n (%) or mean (95 CI%) | *P* value |
|---|---|---|---|
| Training cohort | | | |
| Histological type | | | 0.895 |
| Undiferentiated type | 28 (96.6) | 98 (97.0) | |
| Poorly diferentiated | 1 (3.4) | 3 (3.0) | |
| RBC ($10^{12}$/L) | 4.21 (3.99 - 4.42) | 4.90 (4.78 - 5.01) | < 0.001 |
| B cells ($10^{9}$/L) | 0.11 (0.08 - 0.15) | 0.18 (0.15 - 0.21) | 0.002 |
| NK cells ($10^{9}$/L) | 0.36 (0.26 - 0.46) | 0.31 (0.28 - 0.35) | 0.850 |
| Treg cells | 0.09 (0.07- 0.11) | 0.17 (0.15 - 0.19) | < 0.001 |
| APOB (g/L) | 0.94 (0.85- 1.02) | 0.97 (0.93- 1.01) | 0.480 |
| LDH (U/L) | 241.6 (203.3- 279.9) | 193.0 (180.4- 205.7) | 0.022 |
| LSR | 1.59 (0.90- 2.28) | 1.01 (0.92- 1.09) | 0.062 |
| | | | |
| Validation cohort | | | |
| Histological type | | | 0.018 |
| Undiferentiated type | 15 (88.2) | 46 (100.0) | |
| Poorly diferentiated | 2 (11.8) | 0 (0.0) | |
| RBC ($10^{12}$/L) | 4.08 (3.80- 4.36) | 4.94 (4.70- 5.181) | < 0.001 |
| B cells ($10^{9}$/L) | 0.09 (0.05 - 0.13) | 0.17 (0.13 - 0.20) | 0.011 |
| NK cells ($10^{9}$/L) | 0.38 (0.27 - 0.48) | 0.31 (0.25 - 0.37) | 0.180 |
| Treg cells | 0.11 (0.08 - 0.13) | 0.17 (0.13 - 0.21) | 0.042 |
| APOB (g/L) | 0.99 (0.91 - 1.08) | 1.02 (0.95 - 1.09) | 0.880 |
| LDH (U/L) | 352.4 (51.3- 653.6) | 201.1 (181.7- 220.6) | 0.800 |
| LSR | 1.22 (0.87- 1.58) | 1.06 (0.93- 1.19) | 0.510 |
